# Socioeconomic disparities in behavioral risk factors and health outcomes by gender in the Republic of Korea

**DOI:** 10.1186/1471-2458-10-195

**Published:** 2010-04-15

**Authors:** Hak-Ju Kim, Jennifer Prah Ruger

**Affiliations:** 1 Dongguk University, Department of Social Welfare, Seoul, Republic of Korea; 2 Yale University Schools of Medicine and Public Health, Division of Health Policy and Administration, New Haven, CT, USA

## Abstract

**Background:**

Few studies have examined socioeconomic disparities in health and behavioral risk factors by gender in Asian countries and in South Korea, specifically. We investigated the relationship between socioeconomic position (education, income, and occupation) and subjective and acute and chronic health outcomes and behavioral risk factors by gender, and compared results from 1998 and 2005, in the Republic of Korea.

**Methods:**

We examined data from a nationally representative stratified random sample of 4213 men and 4618 women from the 1998 Korea National Health and Nutrition Examination Survey, and 8289 men and 8827 women from the 2005 Korea National Health and Nutrition Examination Survey using General Linear Modeling and multiple logistic regression methods.

**Results:**

Controlling for behavioral risk factors (smoking, drinking, obesity, exercise, and sleep), those in lower socioeconomic positions had poorer health outcomes in both self-reported acute and chronic disease and subjective measures; differences were especially pronounced among women. A socioeconomic gradient for education and income was found for both men and women for morbidity and self-reported health status, but the gradient was more pronounced in women. In 1998, the odds ratios (ORs) of higher morbidity for illiterate vs. college educated females was 5.4:1 and 1.9:1 for females in the lowest income quintile vs. the highest. The OR for education decreased in 2005 to 2.9:1 and that for income quintiles remained the same at 1.9:1. The OR of lower self-reported health status for illiterate vs. college educated females was 2.9:1 and 1.6:1 for females in the lowest income quintile vs. the highest in 1998, and 3.3:1 and 2.3:1 in 2005.

**Conclusions:**

Among Korean adults, men and women in lower socioeconomic position, as denoted by education, income, and somewhat less by occupation, experience significantly higher levels of morbidity and lower self-reported health status, even after controlling for standard behavioral risk factors. Disparities were more pronounced for women than for men. Efforts to reduce health disparities in South Korea require attention to the root causes of socioeconomic inequality and gender differences in the impact of socioeconomic position on health.

## Background

Health disparities research has documented the significant relationship between social and economic inequalities and health status, although the role of specific types of social and economic stratification (income [1,2], education [3-6], occupation [4-6]) and the role of behavioral risk factors remains an open question [7-12]. National studies differ in their results: a study of European countries found that health inequalities relate to social variables such as education level and occupational class [4]; other studies indicate that health inequalities do not necessarily relate to behavioral factors [3], but to economic disparities such as poverty level [9,10]. One study equated poverty with shorter life expectancy [7]. Further studies give great import to the social environment's impact on health [9,13], especially social class. A comparison of coronary heart disease (CHD) among women from high and low social classes in the UK, for example, found that the latter were 2.7 times more likely to develop CHD than the former [14].

Although concerns over health inequality have grown recently, studies on socioeconomic inequalities in health in Asia, and particularly in the Republic of Korea, are limited [5,6,15]. Furthermore, previous studies of education, occupation and income on health behaviors have produced mixed results [6,15-24]. One study found that education level was related to mortality [6], another found that mortality across education levels remained unchanged over a ten-year period [17], while another found no educational differences in mortality from coronary artery disease for women aged 55 to 64, while mortality in those aged 35 to 54 is inversely related to education level [22], as found in Western Europe and the U.S. Furthermore, previous work varies in the consideration of behavioral risk factors, which could play a significant role in health inequalities [15]. Studies exploring the role of behavioral risk on the relationship between body mass index (BMI) and mortality, for example, provide inconsistent results [25-29]. Some researchers find a strong J-shape or U-shape correlation between BMI and mortality [26-28], whereas a recent study showed that the role of BMI was statistically insignificant, after adjusting for income level [29]. A common limitation is that previous studies have typically focused on either subjective health measures or self-reported acute and chronic disease; few have studied both simultaneously, and fewer still have done so taking gender and behavioral risk factors into account [3,30] and employing data from nationally representative surveys. Conflicting findings from previous studies [6,21,22], combined with limited scope and study populations, demonstrate the need to examine simultaneously, in a national probability sample, various social and economic measures [31-34], behavioral risk factors [35,36], and acute and chronic disease and subjective health measures [34,37] among both men and women in studying health inequality [14,16,24,38-40]. Few studies have focused on how gender differences affect the impact of socioeconomic position on health [22-24].

To our knowledge, this is the first study in South Korea to examine socioeconomic inequalities by three different indicators (education, occupation, and income) in both self-reported acute and chronic disease and subjective health conditions among men and women respectively, controlling for behavioral risk factors (smoking, drinking, obesity, exercise, and sleep), marital status and age. This study tests whether socioeconomic health inequalities differ by: (i) gender; (ii) self-reported or self-rated (subjective) health measures; or (iii) socioeconomic indicators (income, occupation, or education) and whether these associations between socioeconomic positions and health inequality persist over time.

## Methods

### Data

Data for this study are from the Korea National Health and Nutrition Examination Survey (KNHANES), conducted in 1998 and in 2005. The KNHANES is a nationally representative survey sponsored by the Korean Ministry of Health and Welfare and consists of four parts: the Health Interview Survey (HIS), the Health Examination Survey (HES), the Health Behavior Survey (HBS) and the Nutrition Survey (NS). The HIS and HBS contain data estimating the national prevalence of selected diseases and risk factors. The survey procedure consisted of personal household interviews comprising questions on morbidity, activity levels, medical care and health behavior. The HES estimates national population reference distributions of selected health parameters, including pulse rate, blood pressure, cholesterol, triglycerides, fasting blood sugar, hemoglobin, blood urea nitrogen (BUN), creatinine, serum glutamic oxaloacetic transaminase (SGOT), serum glutamine pyruvic transaminase (SGPT), and hepatitis B antigen and antibody. The NS collects information on dietary practices. Overall, the KNHANES collects detailed information on health status, health care use and expenses, and health insurance coverage. For this study's purposes, the respondents included men and women aged 30 and over who participated in the HBS. Adults under 30, who perhaps had not yet completed their education and acquired income or employment, were excluded.

The KNHANES uses a stratified multistage probability sampling design. Because each sample in the survey was not equally selected and involved stratification and clustering, our study required a sampling weight for unbiased national estimates; all estimates in the text and tables have been weighted accordingly. For the 1998 survey, a total number of 12283 Korean households with 39060 household members of all ages took part in the main interview, out of a total sample of 43682 eligible persons, yielding a response rate of 89.4% (HBS: 9808 persons from 13218 persons aged 10 and over with a response rate of 74.2%; HES: 9771 persons from 13420 persons aged 10 and over with a response rate of 72.8%; NS: 11525 persons from 15475 persons with a response rate of 74.5%). For the 2005 survey, 25207 persons out of 25453 completed the main HIS interview with a person-level response rate of 99.1% (HBS: 8835 persons from 9516 persons with a response rate of 92.8%; HES: 7597 persons from 10816 persons with a response rate of 70.2%; NS: 9047 persons from 11240 persons with a response rate of 80.5%). Most questions from the 1998 and 2005 survey are the same, while some were modified slightly or added to the 2005 survey. Among the particular variables we used for this study, the behavioral risk factors are in two- or three-point scale variables to allow equal comparison.

### Self-reported Acute and Chronic Disease and Self-rated Health Outcome Measures

We used morbidity as the self-reported acute and chronic measure and self-rated health condition as the subjective measure for this study. To measure morbidity, we recorded the respondent's presence or absence of illness (all acute and chronic diseases), a value of 0 or 1. Self-rated health status contained five categories: (1) very good; (2) good; (3) average; (4) poor; and (5) very poor.

### Independent Variables

#### Measures of Socioeconomic Position

Three variables identified socioeconomic position: household income, education, and occupation. For household income, we categorized individuals into income quintiles: (1) lowest; (2) low; (3) medium; (4) high; and (5) highest. The lowest quintile is the 20% of the population with the lowest household equivalent income in 1998. Each successive group is the fifth of the population with the next highest household equivalent income. To determine household equivalent income, which reflects total income and the number of adults and children in the household, we used the Organisation for Economic Co-operation and Development (OECD) Equivalence Scale, which equals (1 + 0.7(N_a_-1) + 0.5N_c_), where N_a _= number of adults and N_c _= number of children. This scale is a standard measure for international comparisons of poverty and income inequality [41].

We identified five education levels: (1) illiterate (no formal education); (2) elementary (1-6 years); (3) middle (7-9 years); (4) high (10-12 years); and (5) college and above (>12 years). The occupation variables contained 13 categories: (1) legislator, senior officials, and managers; (2) professionals; (3) technicians and associate professionals; (4) clerks; (5) service and sales workers; (6) skilled agricultural, forestry and fishery workers; (7) craft and related trades workers; (8) plant, machine operators, and assemblers; (9) manual laborers; (10) military personnel; (11) students; (12) housewives; and (13) unemployed. The occupation variables were collapsed into an occupational index of six occupational classes (Class I-VI) (See Additional file 1).

#### Other Sociodemographic Indicators and Behavioral Risk Factors

Personal characteristics for the study included marital status, gender, and age. Marital status fell into three groups: never married, married, and single. Single also included widow or divorced. The behavioral risk factors included smoking (currently smoke, previously smoked, never smoked), alcohol consumption (heavy drinking: drink frequently; moderate drinking: drink once in a while; no drinking: hardly or never drink), weight (obese: BMI [weight (kg)/height (m^2^)] ≥ 25; not obese: BMI < 25), self-reported exercise, and sleep (adequate sleep: ≥ 7 hours daily; inadequate sleep: < 7 hours daily). Previous studies note the use of a lower BMI cut-off point for defining overweight in Asians -- including Koreans -- compared with whites when identifying different risks [42-44]. However, the question on heavy drinking was excluded in the 2005 data. Thus, we used an alternative question which is close to its connotation: "how often do you drink more than 7 cups of Korean hard liquor (soju) or 5 cans of beer at a time?" The original answer scales ranged from no drinking (not applicable) to drinking almost everyday (no drinking: never drink; moderate drinking: drink 7 cups of soju or 5 cans of beers at one spot less than once a month; heavy drinking: drink 7 cups or soju or 5 cans of beers at one spot at least once a month).

### Statistical Methods

We used Analysis of Covariance (ANCOVA) to assess behavioral risk factors by socioeconomic position and then the General Linear Model to compute age-adjusted mean values of health, controlling for respondents' personal characteristics and behavioral risk factors. We analyzed men and women separately for both morbidity and self-rated health. We estimated odds ratios, means, and 95% confidence intervals with multiple logistic regression methods, adjusting for age and other sociodemographic variables. The odds calculated from logistic regressions approximate the relative risk of ill health and morbidity respectively. The p-value for the threshold of significance was set at the 0.01 level. SAS [45] and Excel statistical software were used for all analyses. Sample weights were included for all analyses.

## Results

### Study Population and Sociodemographic Indicators

Table 1 and Table 2 show the 1998 and 2005's distribution of sociodemographic indicators overall and by income quintile, including gender, age, occupation, education, and marital status. The sample included an equal distribution of males (48.2% in 1998, 50.6% in 2005) and females (51.9% in 1998, 49.4% in 2005). Genders were equally distributed across all five income quintiles in both years. In age, 37.0% of the sample was 30-39 and 10.6% was 60-64, with little percentage change in each age bracket from 1998 to 2005 (35.8% in age group 30-39 and 8.1% in age 60-64 in 2005). However, a significant change was found in the income distribution: in 1998, 43.6% of respondents aged 60-64 had the lowest income, while respondents aged 40-49 comprised the greatest percentage (22.5%) of the highest income group in 2005. A small percentage of the overall population was illiterate (5.9% in 1998 and 2.1% in 2005), while a slightly higher percentage (18%) had a college or above education (18.1% in 1998 and 29.1% in 2005). In 1998 and 2005 respectively, the highest percentage of people with elementary education (33.3%, 37.6%) and those illiterate (53.8%, 55.4%) were in the lowest income class. 88.2% of the 1998 sample and 82.3% in 2005 was married. 43.0% of never-married respondents were in the lowest income class in 1998 compared to 23.4% in 2005. In terms of occupation, 21.3% were housewives, 18.0% service and sales workers and 15.1% were skilled agricultural, forestry and fishery workers in 1998. However, the percentage of those in agriculture, forestry and fishery decreased significantly to 5.4% in 2005. Individuals in the lowest income class include the unemployed (47.5% in 1998 and 50.4% in 2005), skilled agriculture, forestry, and fishery workers (35.3% in 1998 and 37.4% in 2005), and manual laborers (27.9% in 1998 and 29.2% in 2005), whereas individuals in the highest income class included legislators, senior officials and managers (39.3% in 1998 and 63.7% in 2005), and professionals (32.9% in 1998 and 57.9% in 2005).

**Table 1:** Sociodemographic Indicators Overall and by Income Quintile (1998)

Indicators	All (%)	Lowest	Low	Medium	High	Highest
**Sex**						

Male	48.15	17.93	19.77	20.57	20.64	21.09

Female	51.85	21.93	20.19	19.48	19.39	19.00

**Age**						

30-39	36.96	12.56	21.24	21.83	22.05	22.32

40-49	30.13	15.47	20.40	22.45	20.79	20.90

50-59	22.34	27.28	19.06	17.26	18.09	18.31

60-64	10.57	43.60	16.43	12.48	14.53	12.95

**Occupation**						

Legislator, senior officials and manager	0.47	0.00	0.00	10.71	50.00	39.29

Professionals	3.46	1.93	6.28	16.91	42.03	32.85

Technicians and associate professionals	2.79	2.40	17.96	22.75	32.93	23.95

Clerks	7.95	2.73	14.08	26.47	32.98	23.74

Service and sales workers	18.00	11.60	20.78	23.84	19.94	23.84

Skilled agricultural, forestry and fishery	15.08	35.33	18.49	12.85	16.72	16.61

Craft and related trades workers	8.25	9.51	25.51	23.89	19.03	22.06

Plant, machine operators and assemblers	4.41	8.71	30.68	21.59	13.64	25.38

Manual laborer	9.40	27.89	19.89	19.18	17.05	15.99

Military personnel	0.07	0.00	0.00	25.00	50.00	25.00

Students	0.10	16.67	33.33	16.67	0.00	33.33

Housewives	21.31	20.14	22.49	22.10	17.95	17.32

Unemployed	8.72	47.51	16.86	10.73	11.69	13.22

**Education**						

Illiterate	5.93	53.80	14.65	11.27	10.42	9.86

Elementary	21.44	33.26	19.47	15.26	14.95	17.06

Middle	18.55	20.43	23.76	20.88	17.37	17.55

High	35.94	13.99	22.03	22.54	19.42	22.03

College & above	18.14	4.79	14.46	22.56	32.87	25.32

**Marital status**						

Never married	7.70	42.95	17.57	15.84	11.28	12.36

Married	88.24	17.52	20.27	20.74	20.86	20.61

Single	4.06	30.45	18.52	11.93	17.70	21.40

Calculations subject to rounding errors

**Table 2:** Sociodemographic Indicators Overall and by Income Quintile (2005)

Indicators	All (%)	Lowest	Low	Medium	High	Highest
**Sex**						

Male	50.58	18.53	19.54	20.45	20.37	21.12

Female	49.42	21.49	20.47	19.55	19.63	18.87

**Age**						

30-39	35.76	15.73	18.99	22.11	21.93	21.23

40-49	34.54	17.32	18.91	20.13	21.18	22.46

50-59	21.62	23.48	22.09	18.03	17.97	18.42

60-64	8.08	39.26	23.12	15.8	12.56	9.26

**Occupation**						

Legislator, senior officials and manager	1.63	1.63	5.83	10.98	17.89	63.69

Professionals	4.32	4.38	7.12	9.06	21.56	57.87

Technicians and associate professionals	4.55	6.98	12.21	17.53	26.07	37.22

Clerks	9.73	3.48	12.22	22.15	25.87	36.28

Service and sales workers	17.61	15.52	19.94	21.1	24.28	19.17

Skilled agricultural, forestry and fishery	5.42	37.4	26.44	13.08	12.04	11.04

Craft and related trades workers	8.93	12.55	23.9	23.68	23.38	16.49

Plant, machine operators and assemblers	6.91	14.98	22.94	27.92	20.49	13.67

Manual laborer	10.16	29.24	27.57	20.32	15.95	6.91

Military personnel	0.42	0	0	27.72	22.28	50

Students	0.22	38.1	10.47	20.95	20.95	9.52

Housewives	21.75	19.86	20.58	21.98	19.75	17.83

Unemployed	8.35	50.43	20.42	12.85	9.65	6.64

**Education**						

Illiterate	2.13	55.41	20.29	12.24	7.71	4.36

Elementary	12.68	37.6	26.55	15.42	13.7	6.73

Middle	13.69	26.71	24.58	20.47	18.47	9.77

High	42.27	17.39	21.69	23.43	20.61	16.87

College & above	29.13	8.76	11.73	17.47	24.06	37.97

**Marital status**						

Never married	8.22	23.39	16.52	18.08	19.04	22.97

Married	82.25	17.76	19.84	20.82	20.64	20.94

Single	9.53	35.6	23.95	14.88	15.57	10

Calculations subject to rounding errors

### Behavioral Risk Factors by Socioeconomic Position

#### Educational Disparities

Table 3 and 4 present age-adjusted education disparities in behavioral risk factors by gender. In terms of obesity, women in the elementary group had the highest rates of obesity (41.5%), while women in the college and above group had the lowest obesity rate (16.5%) in 1998. In 2005, more educated women had the highest rates of obesity (49.5% in high and 54.2% in college and above) compared to illiterate (29.4%) who had the lowest obesity rate. In 1998, the obesity rate for males was highest among the college and above education class (32.9%) and lowest among illiterate respondents (16.0%). In 2005, obesity rates in males were lowest in illiterate (19.7%) and increased to 41.4% in college and above.

**Table 3:** Age-adjusted Educational Disparities in Behavioral Risk Factors by Gender (1998)

Male							Female					
	All (%)	Illiterate	Elemen	Middle	High	College & above	All (%)	Illiterate	Elemen	Middle	High	College & above
**Smoking**												
Never	16.06	10.01	18.19	15.73	13.68	19.33	93.46	89.58	94.48	93.54	94.36	94.05
Ex	19.60	24.19	16.38	19.90	18.86	18.95	1.93	2.07	0.98	1.78	2.29	3.21
Current	64.34	65.81	65.43	64.37	67.47	61.72	4.61	8.35	4.53	4.69	3.35	2.73
	χ2 = 466.54(p < .01, df = 8)						χ2 = 587.01(p < .01, df = 8)					
**Alcohol**												
No	29.00	37.62	28.52	30.41	27.41	24.99	71.79	80.38	76.91	64.08	65.24	70.51
Moderate	35.14	18.76	32.71	31.98	37.71	41.32	24.19	16.73	20.28	29.88	29.47	27.22
Heavy	35.87	43.62	38.77	37.61	34.88	33.70	4.03	2.88	2.80	6.04	5.28	2.26
	χ2 = 596.69(p < .01, df = 8)						χ2 = 1526.23(p < .01, df = 8)					
**Obesity**												
No	71.07	84.04	73.77	72.89	70.00	67.13	68.92	68.06	58.53	61.29	79.08	83.52
Obesity	28.93	15.96	26.23	27.11	30.00	32.87	31.08	31.94	41.47	38.71	20.92	16.48
	χ2 = 263.40(p < .01, df = 4)						χ2 = 3988.73(p < .01, df = 4)					
**Exercise**												
No	78.01	91.81	86.55	79.73	78.91	73.04	83.67	94.71	90.68	83.10	79.78	77.87
Regular	21.99	8.19	13.45	20.27	21.09	26.96	16.33	5.29	9.32	16.90	20.22	22.13
	χ2 = 944.41(p < .01, df = 4)						χ2 = 1831.18(p < .01, df = 4)					
**Sleeping**												
Not	34.17	37.72	34.04	35.49	31.37	34.64	41.26	47.01	45.58	38.23	36.26	35.13
Adequate	65.83	67.28	65.96	64.51	68.63	65.38	58.74	52.99	54.42	61.77	63.74	64.87
	χ2 = 100.02(p < .01, df = 4)						χ2 = 681.25(p < .01, df = 4)					

**Table 4:** Age-adjusted Educational Disparities in Behavioral Risk Factors by Gender (2005)

Male							Female					
	All (%)	Illiterate	Elemen	Middle	High	College & above	All (%)	Illiterate	Elemen	Middle	High	College & above
**Smoking**												
Never	13.85	12.62	11.06	15.71	9.24	18.50	90.27	91.55	91.99	88.75	91.22	94.26
Ex	34.43	30.87	28.17	31.62	28.67	31.66	3.96	0.51	1.85	4.14	3.05	3.13
Current	51.73	56.51	60.77	52.67	62.09	49.84	5.77	7.94	6.16	7.10	5.72	2.61
	χ2 = 1445.77(p < .01, df = 8)						χ2 = 576.25(p < .01, df = 8)					
**Alcohol**												
No	15.24	17.74	18.67	13.83	9.66	9.75	36.03	37.64	33.05	23.47	24.22	29.24
Moderate	13.26	13.81	10.54	11.14	14.79	13.36	32.49	27.76	33.58	35.59	35.12	40.05
Heavy	71.50	68.45	70.79	75.03	75.57	76.89	31.48	34.60	33.38	40.94	40.66	30.71
	χ^2 ^= 577.34(p < .01, df = 8)						χ^2 ^= 1030.22(p < .01, df = 8)					
**Obesity**												
No	62.87	80.23	71.97	59.26	60.10	58.59	67.80	70.64	67.36	52.81	50.51	45.77
Obesity	37.13	19.67	28.03	40.74	39.90	41.41	32.20	29.36	32.64	47.19	49.49	54.23
	χ^2 ^= 258.29(p < .01, df = 4)						χ^2 ^= 3614.32(p < .01, df = 4)					
**Exercise**												
No	50.65	61.68	65.52	60.77	53.85	42.33	56.39	70.64	67.36	52.81	50.51	45.77
Regular	49.35	38.32	34.48	39.23	46.15	57.67	43.61	29.36	32.64	47.19	49.49	54.23
	χ^2 ^= 1719.36(p < .01, df = 4)						χ^2 ^= 1651.81(p < .01, df = 4)					
**Sleeping**												
Not	40.74	34.11	37.86	36.48	39.38	43.63.	42.42	43.34	46.55	41.30	38.63	35.03
Adequate	59.26	65.89	62.14	63.52	60.62	56.37	57.58	56.66	53.45	58.70	61.37	64.97
	χ^2 ^= 209.37(p < .01, df = 4)						χ^2 ^= 438.62(p < .01, df = 4)					

For exercise, male respondents in the highest education classes (27.0% college and above and 21.1% in high) reported exercising on a regular basis in 1998. However, 91.8% of males and 94.7% of females in the lowest education class (illiterate) reported no exercise in 1998. Exercise rates increased in 2005, with 49.4% of all males and 43.6% of all females exercising regularly, especially among illiterate (38.3%) and elementary (34.5%) men and all educational levels of women (29.4% illiterate; 54.2% college and above).

From 1998 to 2005, males became more likely to be obese (28.9% compared to 37.1%), yet also more likely to exercise on a regular basis (22.0% compared to 49.4%).

For smoking, for females, the proportion of people who never smoked was higher in the high and college and above education classes (94.4% and 94.1% in 1998, 91.2% and 94.3% in 2005), but was only slightly lower in the illiterate class (89.6% in 1998 and 91.6% in 2005). Furthermore, females in higher education classes (high and college and above) tended to have lower rates of current smoking (3.4% and 2.7%) in 1998; however, the rates increased to 5.7% and decreased to 2.6%, respectively in 2005. For males, the prevalence of current smoking among the illiterate (65.8%) was higher than that among the college and above education class (61.7%) in 1998. A similar trend was observed in 2005 with 56.5% illiterate current smokers and 49.8% college and above smokers. For alcohol consumption, a higher percentage of males (35.9%) than females (4.0%) reported heavy drinking in 1998; the gender gap persisted in 2005, although heavy drinking increased (71.5% in males and 31.5% in females). In 1998, heavy drinking was more prevalent among illiterate (43.6%) and elementary-educated males (38.8%) as compared to college and above (33.7%) and high-level educated (34.9%) males. For both men and women, heavy drinking increased in 2005 in all the education categories and college and above educated individuals drank more heavily among men but not women. For women, the prevalence of heavy drinking in 1998 was highest in the middle and high educated groups (6.0% and 5.3%) and lowest in the college and above educated (2.3%) and elementary (2.8%) groups.

In terms of adequate sleep, there were no major differences by education in sleeping habits for males, though the result was statistically different; for females the lowest rates of adequate sleep were in the illiterate and elementary education classes (53.0% and 54.4% respectively) in 1998; the pattern was more or less the same in 2005.

#### Income Disparities

Table 5 and 6 report age-adjusted income disparities in behavioral risk factors by gender. In 1998, over four times as many women in the lowest income group (8.1%) reported currently smoking compared to women in the highest income group (1.7%). In 2005, current smoking rates declined to 4.5% in the lowest income group but increased to 8.2% in the highest income group. Overall, women reporting current smoking increased from 4.6% in 1998 to 5.8% in 2005. The average current smoking rate for men dropped overall from 64.3% in 1998 to 51.7% in 2005. But the income gradient remained, as the lowest and low income groups had the highest current smoking rates in both 1998 (69.9% and 64.6%) and 2005 (64.7% and 75.4%) compared to all other income groups.

**Table 5:** Age-adjusted Income Disparities in Behavioral Risk Factors by Gender (1998)

Male							Female					
	All	Lowest	Low	Medium	High	Highest	All (%)	Lowest	Low	Medium	High	Highest
	(%)	(20%)	(20%)	(20%)	(20%)	(20%)		(20%)	(20%)	(20%)	(20%)	(20%)
**Smoking**												
Never	16.06	13.39	15.47	16.30	17.00	17.55	93.46	89.08	93.11	94.60	95.56	96.77
Ex	19.60	16.66	19.92	19.42	18.84	18.72	1.93	2.83	2.73	1.89	1.13	1.56
Current	64.34	69.94	64.61	64.28	64.17	63.73	4.61	8.09	4.16	3.51	3.31	1.67
	χ2 = 201.71(p < .01, df = 8)						χ2 = 1197.22(p < .01, df = 8)					
**Alcohol**												
No	29.00	29.08	26.38	29.08	26.44	26.81	71.79	72.08	66.26	69.36	39.56	69.04
Moderate	35.14	35.82	36.17	35.74	37.93	38.63	24.19	22.47	28.95	26.64	25.80	28.18
Heavy	35.87	35.10	37.45	35.18	35.63	34.55	4.03	5.45	4.78	4.00	4.64	2.79
	χ2 = 102.83(p < .01, df = 8)						χ2 = 371.57(p < .01, df = 8)					
**Obesity**												
No	71.07	74.50	74.97	70.25	71.37	62.32	68.92	70.20	67.14	70.38	73.22	73.26
Obesity	28.93	25.50	25.03	29.75	28.63	37.68	31.08	29.80	32.86	29.62	26.78	26.74
	χ2 = 715.82(p < .01, df = 4)						χ2 = 208.62(p < .01, df = 4)					
**Exercise**												
No	78.01	80.75	83.20	76.06	75.12	78.28/	83.67	88.58	84.06	80.98	80.80	83.28
Regular	21.99	19.25	16.80	23.94	24.88	21.72	16.33	11.42	15.94	19.02	19.20	16.72
	χ2 = 444.66(p < .01, df = 4)						χ2 = 493.38(p < .01, df = 4)					
**Sleeping**												
Not	34.17	35.72	27.08	36.30	32.03	35.60	41.26	41.30	39.69	36.13	38.04	40.96
Adequate	65.83	64.28	72.923	63.70	67.97	64.40	58.74	58.70	60.31	63.87	61.96	59.04
	χ2 = 458.29(p < .01, df = 4)						χ2 = 138.00(p < .01, df = 4)					

**Table 6:** Age-adjusted Income Disparities in Behavioral Risk Factors by Gender (2005)

Male							Female					
	All	Lowest	Low	Medium	High	Highest	All (%)	Lowest	Low	Medium	High	Highest
	(%)	(20%)	(20%)	(20%)	(20%)	(20%)		(20%)	(20%)	(20%)	(20%)	(20%)
**Smoking**												
Never	13.85	10.46	11.57	11.97	13.09	14.66	90.27	92.66	95.72	89.02	80.52	87.93
Ex	34.43	24.87	13.05	28.05	25.46	34.05	3.96	2.83	1.97	2.95	8.03	3.87
Current	51.73	64.67	75.37	59.98	61.45	51.30	5.77	4.51	2.31	8.03	11.44	8.20
	χ^2 ^= 1178.43(p < .01, df = 8)						χ^2 ^= 2021.92(p < .01, df = 8)					
**Alcohol**												
No	15.24	15.52	11.69	17.32	12.12	9.09	36.03	25.62	30.16	26.08	20.68	25.08
Moderate	13.26	18.38	15.84	19.04	11.73	13.24	32.49	35.67	37.10	35.25	39.26	31.05
Heavy	71.50	66.10	72.48	63.68	76.15	77.67	31.48	38.71	32.69	38.67	40.06	43.87
	χ^2 ^= 788.93(p < .01, df = 8)						χ^2 ^= 540.24(p < .01, df = 8)					
**Obesity**												
No	62.87	58.41	71.52	60.44	64.66	58.07	67.80	71.77	72.25	70.06	68.37	70.11
Obesity	37.13	41.59	28.48	39.56	35.34	41.93	32.20	28.23	27.75	29.94	31.63	29.89
	χ^2 ^= 221.84(p < .01, df = 4)						χ^2 ^= 39.36(p < .01, df = 4)					
**Exercise**												
No	50.65	55.19	58.16	60.94	58.43	45.40	56.39	54.71	48.55	50.71	61.91	54.99
Regular	49.35	44.81	41.84	39.06	41.57	54.60	43.61	45.29	51.45	49.29	38.09	45.01
	χ^2 ^= 1244.23(p < .01, df = 4)						χ^2 ^= 469.30(p < .01, df = 4)					
**Sleeping**												
Not	40.74	35.25	48.87	42.74	39.42	40.78	42.42	36.58	38.21	40.26	44.01	50.11
Adequate	59.26	64.75	51.13	57.26	60.58	59.22	57.58	63.42	61.79	59.74	55.99	49.89
	χ^2 ^= 122.68(p < .01, df = 4)						χ^2 ^= 415.16(p < .01, df = 4)					

In alcohol consumption in 1998, men and women in the lowest income group had higher rates of heavy drinking than those in the highest income group (35.1% to 34.6% for men, 5.5% to 2.8% for women). This trend reversed in 2005, when men and women in the highest income group had higher rates of heavy drinking than those in the lowest income group (77.7% to 66.1% for men, 43.9% to 38.7% for women).

For obesity rates in 1998, women in the lowest income group had higher rates of obesity compared to women in the highest class (29.8% to 26.7%) while men in the lowest income group had lower rates of obesity compared to men in the highest class (25.5% to 37.7%).

Compared to 1998, more men and women in all income groups reported exercising regularly, but the gap between regularly exercising in the lowest and highest groups by income increased in 2005 in men (2.5% difference in 1998, 9.8% difference in 2005) but decreased in women (5.3% difference in 1998, 0.3% difference in 2005). In 1998 the greatest percentage of females in the lowest income class reported inadequate sleep (41.3%) while in 2005, the greatest percentage of females in the highest income class reported inadequate sleep (50.1%).

#### Occupational Disparities

Table 7 and 8 show age-adjusted occupational disparities in behavioral risk factors, using the Korean Occupational Classification Index (see Additional file 1). For women, rates of heavy drinking were higher in the middle classes (Class II-IV) than in both the lowest and highest classes in 1998 and 2005. For men, heavy drinking was much lower for the lowest class (Class I) (27.4% in 1998) compared to all other classes in 1998; in 2005, heavy drinking was much lower in Class III (45.1%) compared to all other classes In 1998, obesity rates were highest in Classes I, II and III occupational class among men (34.8%, 31.4% and 34.6%) and Classes II, III, and IV (41.9%, 39.7%, and 40.6%) in 2005. Obesity rates were highest in Classes III and VI for women in both 1998 and 2005 (36.1% and 40.5% in 1998 versus 36.4% and 34.7% in 2005).

**Table 7:** Age-adjusted Occupational Disparities in Behavioral Risk Factors by Gender (1998)

	Male							Female						
	All (%)	Class I	Class II	Class III	Class IV	Class V	Class VI	All (%)	Class I	Class II	Class III	Class IV	Class V	Class VI
**Smoking**														
Never	16.06	24.41	17.40	13.81	15.48	17.58	13.65	93.46	97.87	93.24	90.18	91.23	96.93	91.57
Ex	19.60	20.14	23.09	15.16	17.79	19.91	14.63	1.93	0.47	3.07	1.77	1.88	1.77	1.95
Current	64.34	55.45	59.51	71.03	66.73	62.51	71.72	4.61	1.66	3.69	8.05	6.89	1.31	6.48
	χ2 = 1002.43(p < .01, df = 10)							χ2 = 312.52(p < .01, df = 10)						
**Alcohol**														
No	29.00	24.18	29.87	25.77	29.63	23.64	24.18	71.79	76.34	59.87	64.63	59.63	78.90	66.27
Moderate	35.14	38.58	39.62	31.23	40.36	30.25	38.14	24.19	23.66	32.28	25.93	33.52	17.41	30.88
Heavy	35.87	27.42	36.19	38.90	33.87	40.12	38.22	4.03	0.00	7.85	9.44	6.85	3.69	2.85
	χ2 = 806.58(p < .01, df = 10)							χ2 = 774.32(p < .01, df = 10)						
**Obesity**														
No	71.07	65.22	68.58	65.38	70.86	75.42	80.23	68.92	88.54	77.85	63.94	74.40	65.20	59.48
Obesity	28.93	34.78	31.42	34.62	29.14	24.58	19.77	31.08	11.46	22.15	36.06	25.60	34.80	40.52
	χ2 = 579.03(p < .01, df = 5)							χ2 = 942.24(p < .01, df = 5)						
**Exercise**														
No	78.01	66.05	73.72	79.08	80.69	89.82	81.99	83.67	83.15	84.46	89.64	88.13	98.23	89.76
Regular	21.99	33.95	26.28	20.92	19.37	10.18	18.01	16.33	16.85	15.54	10.36	11.87	1.77	10.24
	χ2 = 1505.30(p < .01, df = 5)							χ2 = 315.46(p < .01, df = 5)						
**Sleeping**														
No	34.17	39.09	33.32	32.43	34.49	29.97	40.32	41.26	43.78	44.50	50.11	44.94	38.80	55.78
Adequate	65.83	60.97	66.68	67.57	65.51	70.03	59.68	58.74	56.22	55.50	49.89	55.06	61.20	44.22
	χ2 = 256.52(p < .01, df = 5)							χ2 = 297.76(p < .01, df = 5)						

**Table 8:** Age-adjusted Occupational Disparities in Behavioral Risk Factors by Gender (2005)

	Male							Female						
	All (%)	Class I	Class II	Class III	Class IV	Class V	Class VI	All (%)	Class I	Class II	Class III	Class IV	Class V	Class VI
**Smoking**														
Never	13.85	32.82	13.30	11.46	11.49	15.87	10.79	90.27	92.59	93.13	88.04	88.71	98.45	85.54
Ex	34.43	35.46	33.97	27.51	26.70	33.14	22.51	3.96	4.76	2.25	4.04	3.46	0.00	3.19
Current	51.73	31.72	52.73	61.04	61.81	50.99	66.90	5.77	2.64	4.63	7.92	7.84	1.55	11.26
	χ^2 ^= 2722.32(p < .01, df = 10)							χ^2 ^= 464.86(p < .01, df = 10)						
**Alcohol**														
No	15.24	15.90	7.21	11.82	7.20	16.84	9.05	36.03	22.05	21.90	23.23	20.80	27.68	26.15
Moderate	13.26	16.99	13.43	13.06	13.89	12.76	10.63	32.49	37.99	37.96	31.47	33.56	47.38	34.64
Heavy	71.50	67.11	79.36	45.12	78.92	70.39	80.33	31.48	39.97	40.14	45.29	45.64	24.93	39.21
	χ^2 ^= 895.44(p < .01, df = 10)							χ^2 ^= 265.51(p < .01, df = 10)						
**Obesity**														
No	62.87	60.84	58.08	60.31	59.41	61.04	70.25	67.80	83.37	85.62	63.62	73.60	71.13	65.26
Obesity	37.13	39.52	41.92	39.69	40.59	38.96	29.75	32.20	16.63	14.38	36.38	26.40	28.87	34.74
	χ^2 ^= 120.68(p < .01, df = 5)							χ^2 ^= 911.05(p < .01, df = 5)						
**Exercise**														
No	50.65	31.75	43.14	58.22	52.86	67.55	68.22	56.39	43.71	51.18	52.96	66.97	70.40	64.21
Regular	49.35	68.25	56.86	41.78	47.14	32.45	31.78	43.61	56.29	48.82	47.04	33.03	29.60	35.79
	χ^2 ^= 2430.68(p < .01, df = 5)							χ^2 ^= 1065.05(p < .01, df = 5)						
**Sleeping**														
No	40.74	50.34	41.00	35.67	40.36	33.47	49.77	42.42	35.34	42.10	48.09	44.79	37.34	46.48
Adequate	59.26	49.66	59.00	64.33	59.64	66.53	50.23	57.58	64.66	57.90	51.91	55.21	62.66	53.52
	χ^2 ^= 564.98(p < .01, df = 5)							χ^2 ^= 190.18(p < .01, df = 5)						

### Differences in Self-reported Acute and Chronic Disease and Subjective Health Outcomes by Socioeconomic Position

In age-adjusted means and odds ratios (OR) in morbidity (Table 9, 10) and self-reported health (Table 11, 12) after adjusting for personal characteristics and behavioral risk factors, we found statistically significant mean differences in morbidity between different education groups for both males and females, but the gap in morbidity decreased for women in 2005. For example, the OR for higher morbidity was 5.4:1 for illiterate versus college and above level females in 1998, but the difference fell to 3.0:1 in 2005. There exists an education gradient in the odds for higher morbidity among men and women in 1998, which was especially steep among women, and although the educational gradient continues to exist in 2005, the gender gap has reduced. An education gradient was also seen for the odds of lower self-reported health status, although it was not as steep as in the morbidity data, even among women; ORs for lower self-reported health was 2.9:1 for illiterate versus college and above level females (2.2 for men) in 1998, and the ratio increased slightly to 3.3:1 (2.7 for men) in 2005.

**Table 9:** Age-adjusted Means* and Odds Ratios** of Morbidity among Korean Men and Women by Socioeconomic Position (1998)

Social class	Lower ←---------------------------------------------------------------------------------→ Higher						
**Education**	**Illiteracy**	**Elementary**	**Middle**	**High**	**College or above**		**Sig**

Male	0.913^A^	0.842^A^	0.794^B^	0.764^BC^	0.684^C^		F = 6.62 (p < .01, df = 4)
	
	2.86 (0.99 - 8.30)	1.63 (1.15 - 2.33)	1.38 (1.02 - 1.89)	1.36 (1.08 - 1.73)	1.00		

Female	0.957^A^	0.933^A^	0.819^B^	0.766 ^C^	0.697^C^		F = 16.72 (p < .01, df = 4)
	
	5.37 (2.68 - 10.76)	4.06 (2.67 - 6.20)	1.54 (1.10 - 2.17)	1.35 (1.02 - 1.80)	1.00		

**Income** **Quintile**	**Lowest** **(20%)**	**Low** **(20%)**	**Medium** **(20%)**	**High** **(20%)**	**Highest** **(20%)**		**Sig**

Male	0.807^A^	0.771^AB^	0.764^AB^	0.754^AB^	0.743^B^		F = 6.39 (p < .01, df = 4)
	
	1.17 (0.85 - 1.61)	1.15 (0.85 - 1.54)	1.11 (0.83 - 1.48)	1.05 (0.78 - 1.40)	1.00		

Female	0.907^A^	0.820^BC^	0.852^B^	0.791^C^	0.782^C^		F = 16.90 (p < .01, df = 4)
	
	1.89 (1.33 - 2.69)	1.19 (0.88 - 1.61)	1.61 (1.18 - 2.21)	1.04 (0.78 - 1.40)	1.00		

**Occupation** **Index**	**Class VI**	**Class V**	**Class IV**	**Class III**	**Class II**	**Class I**	**Sig**

Male	0.840^A^	0.848^A^	0.734^B^	0.767^B^	0.716^B^	0.699^B^	F = 5.23 (p < .01, df = 5)
	
	1.49(0.96 -- 2.33)	1.42(0.96 -- 2.08)	1.00(0.79 -- 1.52)	1.09(0.79 -- 1.52)	0.97(0.71 -- 1.32)	1.00	

Female	0.917^AB^	0.973^A^	0.819^B^	0.833^B^	0.640^C^	0.620^C^	F = 7.67 (p < .01, df = 5)
	
	1.46(0.90 -- 2.35)	4.56(1.11 -- 8.85)	0.97(0.69 -- 1.36)	0.94(0.63 -- 1.41)	0.50(0.33 -- 0.74)		1.00

*Age-adjusted means calculated with age adjustment using Generalized Linear Model; **odds ratios (95%CI) computed using logistic regression, controlling for respondents' personal characteristics (age and marital status) and behavioral risk factors (smoking, alcohol, obesity, exercise, sleeping). Socioeconomic position measured by education, income, and occupational class. Highest socioeconomic position in education, income, and occupation index was reference (1.0), respectively. Duncan's post-hoc test is implemented to compare ground differences. Same alphabetical letters indicates no statistical differences between two mean values. Morbidity (0 = lowest, 1 = highest).

**Table 10:** Age-adjusted Means* and Odds Ratios** of Morbidity among Korean Men and Women by Socioeconomic Position (2005)

Social class	Lower ←-------------------------------------------------------------------------------------------→ Higher						
**Education**	**Illiteracy**	**Elementary**	**Middle**	**High**	**College or above**		**Sig**

Male	0.872^A^	0.671^B^	0.664^B^	0.627^B^	0.559^C^		F = 5.89 (p < .01, df = 4)
	
	2.97(1.73 - 5.09)	1.98(1.64 - 2.39)	1.69(1.44 - 1.98)	1.38(1.24 - 1.52)	1.00		

Female	0.669^B^	0.750^A^	0.736^A^	0.648^B^	0.553^C^		F = 10.79 (p < .01, df = 4)
	
	2.96(2.08 - 4.21)	2.70(2.26 - 3.22)	2.33(1.98 - 2.73)	1.41(1.26 - 1.59)	1.00		
**Income** **Quintile**	**Lowest** **(20%)**	**Low** **(20%)**	**Medium** **(20%)**	**High** **(20%)**	**Highest** **(20%)**		**Sig**

Male	0.693^A^	0.645^AB^	0.625^B^	0.617^BC^	0.551^C^		F = 5.76 (p < .01, df = 4)
	
	1.83(1.57 - 2.14)	1.49(1.29 - 1.71)	1.27(1.10 - 1.43)	1.21(1.06 - 1.38)	1.00		

Female	0.735^A^	0.714^AB^	0.675^ABC^	0.632^CD^	0.595^D^		F = 14.36 (p < .01, df = 4)
	
	1.93(1.66 - 2.25)	1.72(1.49 - 1.99)	1.36(1.19 - 1.56)	1.24(1.09 - 1.42)	1.00		

**Occupation** **Index**	**Class VI**	**Class V**	**Class IV**	**Class III**	**Class II**	**Class I**	**Sig**

Male	0.669^A^	0.649^AB^	0.610^BC^	0.646^AB^	0.546^C^	0.535^C^	F = 3.96 (p < .01, df = 5)
	
	1.79(1.41 - 2.28)	1.86(1.42 - 2.43)	1.40(1.16 - 1.70)	1.43(1.17 - 1.74)	1.11(0.92 - 1.35)	1.00	

Female	0.691	0.625	0.691	0.650	0.643	0.394	F = 2.08 (p < .01, df = 5)
	
	2.12(1.56 - 2.89)	3.02(1.62 - 5.62)	2.06(1.56 - 2.72)	1.49(1.10 - 2.02)	1.33(0.99 - 1.78)	1.00	

*Age-adjusted means calculated with age adjustment using Generalized Linear Model; **odds ratios (95%CI) computed using logistic regression, controlling for respondents' personal characteristics (age and marital status) and behavioral risk factors (smoking, alcohol, obesity, exercise, sleeping). Highest socioeconomic position in education, income, and occupation index was referent (1.0), respectively. Duncan's post-hoc test is implemented to compare ground differences. Same alphabetical letters indicates no statistical differences between two mean values. Morbidity (0 = lowest, 1 = highest).

**Table 11:** Age-adjusted Means* and Odds Ratios** in Self-Reported Health among Korean Men and Women by Socioeconomic Position (1998)

Social class	Lower ←-----------------------------------------------------------------------------------→ Higher						
**Education**	**Illiteracy**	**Elementary**	**Middle**	**High**	**College or above**		**Sig**

Male	3.087^A^	2.912^AB^	2.883^B^	2.624^C^	2.466^C^		F = 15.69 (p < .01, df = 4)
	
	2.16 (1.22 - 3.85)	1.72 (1.33 - 2.25)	1.98 (1.55 - 2.52)	1.28 (1.06 - 1.56)	1.00		

Female	3.350 ^A^	3.199^B^	2.898^C^	2.817^C^	2.635^D^		F = 18.19 (p < .01, df = 4)
	
	2.94 (2.07 - 4.18)	2.40 (1.83 - 3.17)	1.53 (1.18 - 1.98)	1.37 (1.09 - 1.73)	1.00		

**Income** **Quintile**	**Lowest** **(20%)**	**Low** **(20%)**	**Medium** **(20%)**	**High** **(20%)**	**Highest** **(20%)**		**Sig**

Male	2.862^A^	2.742^B^	2.647^BC^	2.596^C^	2.631^BC^		F = 15.18 (p < .01, df = 4)
	
	1.33 (1.04 - 1.69)	1.16 (0.93 - 1.47)	1.02 (0.81 - 1.28)	0.94 (0.74 - 1.17)	1.00		

Female	3.216^A^	2.993^B^	2.907^BC^	2.847^C^	2.841^C^		F = 17.84 (p < .01, df = 4)
	
	1.63 (1.31 - 2.02)	1.32 (1.07 - 1.63)	1.16 (0.94 - 1.44)	0.98 (0.79 - 1.21)	1.00		

**Occupation** **Index**	**Class VI**	**Class V**	**Class IV**	**Class III**	**Class II**	**Class I**	**Sig**

Male	2.901^A^	2.829^A^	2.688^B^	2.662^BC^	2.535^CD^	2.447^D^	F = 12.19 (p < .01, df = 5)
	
	1.25(0.91 -- 1.71)	0.95(0.73 -- 1.26)	1.09(0.85 -- 1.39)	0.97(0.75 -- 1.25)	0.88(0.68 -- 1.13)	1.00	

Female	3.252^A^	3.240^A^	2.693^B^	2.787^B^	2.592^B^	2.775^B^	F = 7.98 (p < .01, df = 5)
	
	1.24(0.97 -- 1.59)	1.14(0.74 -- 1.74)	0.56(0.44 -- 0.71)	0.61(0.46 -- 0.79)	0.56(0.40 -- 0.79)	1.00	

*Age-adjusted means calculated with age adjustment using Generalized Linear Model; **odds ratios (95%CI) computed using logistic regression, controlling for respondents' personal characteristics (age and marital status) and behavioral risk factors (smoking, alcohol, obesity, exercise, sleeping). Highest socioeconomic position in education, income, and occupation was referent (1.0), respectively. Socioeconomic position measured by education, income, and occupational class. Duncan's post-hoc test is implemented to compare ground differences. Same alphabetical letters indicates no statistical differences between two mean values. Self-reported health (5 = very poor, 4 = poor, 3 = average, 2 = good, 1 = very good).

**Table 12:** Age-adjusted Means* and Odds Ratios** in Self-Reported Health among Korean Men and Women by Socioeconomic Position (2005)

Social class	Lower ←---------------------------------------------------------------------------------------→ Higher						
**Education**	**Illiteracy**	**Elementary**	**Middle**	**High**	**College**		**Sig**

Male	3.433^A^	2.853^B^	2.818^B^	2.663^C^	2.539^D^		F = 6.96 (p < .01, df = 4)
	
	2.65(1.67 - 4.18)	2.15(1.80 - 2.57)	1.88(1.61 - 2.20)	1.28(1.15 - 1.41)	1.00		

Female	3.600^A^	3.159^B^	2.974^C^	2.757^D^	2.656^E^		F = 41.66 (p < .01, df = 4)
	
	3.31(2.37 - 4.64)	2.60(2.19 - 3.09)	2.00(1.71 - 2.33)	1.25(1.12 - 1.41)	1.00		

**Income** **Quintile**	**Lowest** **(20%)**	**Low** **(20%)**	**Medium** **(20%)**	**High** **(20%)**	**Highest** **(20%)**		**Sig**

Male	3.019^A^	2.757^B^	2.673^C^	2.612^CD^	2.541^D^		F = 23.34 (p < .01, df = 4)
	
	2.31(1.99 - 2.69)	1.56(1.36 - 1.79)	1.25(1.10 - 1.43)	1.13(0.99 - 1.29)	1.00		

Female	3.161^A^	2.996^B^	2.899^C^	2.823^C^	2.661^D^		F = 26.92 (p < .01, df = 4)
	
	2.30(1.97 - 2.67)	1.77(1.54 - 2.04)	1.36(1.19 - 1.55)	1.16(1.01 - 1.32)	1.00		

**Occupation** **Index**	**Class VI**	**Class V**	**Class IV**	**Class III**	**Class II**	**Class I**	**Sig**

Male	2.796^A^	2.690^AB^	2.649^AB^	2.677^AB^	2.489B	2.523^B^	F = 4.91 (p < .01, df = 5)
	
	1.62(1.28 - 2.06)	1.76(1.36 - 2.28)	1.37(1.13 - 1.66)	1.25(1.03 - 1.53)	1.06(0.87 - 1.29)	1.00	

Female	3.014^A^	3.471^A^	2.825^B^	2.841^B^	2.598^B^	2.439^B^	F = 8.06 (p < .01, df = 5)
	
	2.42(1.78 - 3.29)	4.65(2.46 - 8.77)	1.74(1.33 - 2.29)	1.43(1.06 - 1.93)	1.250(0.93 - 1.66)	1.00	

*Age-adjusted means calculated with age adjustment using Generalized Linear Model; **odds ratios (95%CI) computed using logistic regression, controlling for respondents' personal characteristics (age and marital status) and behavioral risk factors (smoking, alcohol, obesity, exercise, sleeping). Highest socioeconomic position in education, income, and occupation was referent (1.0), respectively. Socioeconomic position measured by education, income, and occupational class. Duncan's post-hoc test is implemented to compare ground differences. Same alphabetical letters indicates no statistical differences between two mean values. Self-reported health (5 = very poor, 4 = poor, 3 = average, 2 = good, 1 = very good).

The income gradient in adjusted odds ratios was not as steep as for education in the 1998 data, although a weak gradient did exist and again was more pronounced among women than men. In 1998, the OR of morbidity from lowest to highest income quintile in women was 1.9:1 and the OR of self-reported health from lowest to highest income quintile in women was 1.6:1. The OR of morbidity from lowest to highest income quintile in men was 1.2:1 and the OR of self-reported health from lowest to highest income quintile was 1.3:1 in 1998. The income gradient in ORs in self-reported health among both men and women increased from 1998 to 2005; the OR for lower self-reported health status increased from 1.3:1 to 2.3:1 for males and from 1.6:1 to 2.3:1 for females.

The findings for occupational classification were less clear cut in 1998; while adjusted odds ratios for the lowest versus the highest class were significant (1.5:1 for morbidity for both males and females and roughly 1.2:1 for self-reported health for both males and females), the gradient did not exist such that those in the second highest class, and in some cases the third highest class, had greater adjusted ORs than the highest occupational class. In 2005, the greatest inequality between the lowest and highest occupational classes occurred between Classes V and I for self reported health (with an OR of 4.7:1 in women and 1.8 in men) and for morbidity (with an OR of 3.0 in women and 1.9 in men).

## Discussion

Substantial research has focused on the relationship between socioeconomic position and health status, although few studies have been conducted in Asian countries. Moreover, health outcome measures used in Korea have largely been limited to either self-reported health status or mortality. Using composite indices, one study found mortality risks for breast and cervical cancer was much greater in lower income groups [46]. Previous studies have also typically targeted a single socioeconomic variable (e.g. education or income) and a common limitation is the inability to analyze behavioral risk factors alongside socioeconomic groupings in a national sample. One Korean study, for example, found that educational gradients in mortality were not consistent across causes of death and in some cases differed by sex [22] whereas another study found that a negative relationship between socioeconomic status and cardiovascular disease among men did not change by introducing cardiovascular risk factors [47]. Using three socioeconomic measures (education, occupation, and income) to capture differences in both self-reported acute and chronic disease and subjective health status, this study found that differences in personal characteristics and behavioral risk factors relate significantly to socioeconomic groups, but that the relationship was not necessarily linear in fashion and there was no clear socioeconomic gradient with behavioral risk factors. The results did, however, confirm an association between socioeconomic position and morbidity [48,49] and self-reported health status. In 1998, health inequalities among socioeconomic groups were significant: age-adjusted mean differences in health outcomes were higher in self-reported health status than for morbidity, findings that are consistent with previous work in India [37]. An education gradient in morbidity was found for both men and women, but was especially steep among women; a weaker education gradient existed for self-reported health, even among women. The income gradient for morbidity and self-reported health status was not as steep as for education, although a weak gradient did exist and was also more pronounced among women. Findings for occupational classification were also statistically significant. Thus, in general, the differences among income groups were smaller than among education groups, suggesting that educational disparities delineate a different socioeconomic gradient from income disparities. In 2005, the education gradient for morbidity decreased significantly among women, approaching the odds ratios for men. Gradients in self-reported health, by contrast, became steeper for both genders in all socioeconomic variables.

The gender implications of this study are significant and require further study. Some studies have found that health inequalities among different income groups affect mortality in women less than in men [11,23,24], while others have found the opposite to be true [14,23,24]. In this study, however, inequalities in both morbidity and self-reported health status were greater, and the socioeconomic gradient in education and income was steeper, among women than men, although the educational gradient had decreased by roughly 40% by 2005. The steeper education and income gradients compared to the relative lack of gradient in occupation, especially among women, require explanation and further study. One explanation may be the more enduring effect of income and education disparities due to the more ingrained social hierarchies in these variables, which prevail from childhood. Occupational disparities, by contrast, commence primarily in adulthood [3,30]. One recent study, for example, explained gender disparities in self-rated health as resulting from Korea's social structure [50].

Another possible explanation points to the relatively lower percentage of Korean women who are employed and participate in regular or professional job markets, compared to their western counterparts and the relative insecurity of employment. This comports with research that analyzed data from the 2001 Korean Labor and Income Survey and found that precarious employment had a significant association with worse self-reported health for women as compared to men [51]. Another study that looked at the 2001 KNHANES found a relationship between non-standard employment (less job security) and health that differed by gender: Korean female non-standard employees had higher rates of mental disorders compared to men whereas males in non-standard employment had higher rates of musculoskeletal disorders and liver disease [52]. As a result, for Korean women, household income could be significantly more important than occupation in determining deprivations in health.

Another explanation, in particular for the gender differential in the impact of socioeconomic position on health that cannot be explained by differences in behavioral risk factors alone, may be women's overall inferior status in Korean society, beginning from birth. Though South Korea is regarded as one of the most advanced countries in Asia, some socioeconomic obstacles for women still remain, especially in the successful pursuit of women's careers and economic wealth and stability. This trend is exacerbated in Korea by the high cost of education, which can create barriers for women in accessing health care, health knowledge and information, as well as preventive screening services.

Although there is growing social awareness of the importance of equal opportunities for and treatment of both genders, implementation of policies designed to promote women's rights are slow and sometimes even halted under the pretext of efficiency and economic development. Although Korea is among the world's top ten economies, it was ranked 108th among 130 countries in the Global Gender Gap report in 2008, leaving only a score of Arab and African countries behind it [53]. In the past, when women mostly engaged in jobs requiring low-level skills, companies did not think of the female labor force as increasing their financial burden. Today, however, businesses are reluctant to fully utilize the female talent pool for fear it will cost them more, as there is increased societal pressure to offer women equal employment opportunities while protecting their motherhood at the same time. When a society restricts women's job opportunities and socioeconomic prospects, it could further harm their self-reported acute and chronic health conditions, making it possible for health disparities to be larger in females than in males.

Gender-specific health differentials may possibly be narrowed by health promotional activities specifically targeted towards women in lower socioeconomic classes. Behavioral risk factors did not entirely account for differences in self-reported health and morbidity among socioeconomic position. This result suggests that choices regarding cigarette smoking, drinking, body weight, exercise, and adequate sleeping may only partly change socioeconomic health inequalities in Asia, although significant differences are found in terms of gender.

This study has some limitations. First, lack of response and missing values for some variables might cause bias, though previous studies of the KNHANES have not detected bias [54,55]. Second, self-reports of household income could be prone to error, despite corroboration by other household members. Third, the personal interviews for the 1998 data took place at the end of 1998, directly after the Asian economic crisis. The crisis might have affected the morbidity rates in both men and women. In addition, some respondents reporting low income levels might previously have earned more and enjoyed better health care.

## Conclusions

This study explored varying impacts of education, occupation, and income on health inequalities in men and women, separately. The results are consistent with those of the Whitehall studies of British civil servants, where social gradients in morbidity existed from the bottom to the top of the hierarchy [7,8,56]. An interesting finding from this study was that socioeconomic differences in health appeared to be higher among women than men. After adjusting for the respondents' behavioral risk factors, the mean differences among socioeconomic classes for both male and female morbidity increased. These results demonstrate that the magnitude of health inequalities can differ by socioeconomic variables and by personal and behavioral risk factors. Education and household income showed the most significant effect on differences among levels, while occupational groups showed little health difference, although odds ratios between groups increased significantly after controlling for behavioral risk factors. Efforts to reduce health disparities in South Korea require attention to the root causes of socioeconomic inequality and especially to the gender differences in the impact of socioeconomic position on health.

## Competing interests

The authors declare that they have no competing interests.

## Authors' contributions

HK contributed to the conception and design of this study, undertook data analyses and interpretation and wrote the original draft. JR contributed to the conception and design of the study and contributed to revising the manuscript. All authors have read and approved the final manuscript.

## Pre-publication history

The pre-publication history for this paper can be accessed here:

http://www.biomedcentral.com/1471-2458/10/195/prepub

## Supplementary Material

Additional file 1 **Appendix A**. Korean Occupational Classification Index. Click here for file
