# Supplementary material for: Socioeconomic disparities in behavioral risk factors and health outcomes by gender in the Republic of Korea
Source: BMC Public Health. 2010 Apr 15;10:195. doi: 10.1186/1471-2458-10-195 (PMC2867999; doi:10.1186/1471-2458-10-195)
Supplement: Additional file 1 — Appendix A. Korean Occupational Classification Index. [file 1471-2458-10-195-S1.DOCX]

**Appendix A:** Korean Occupational Classification Index

| Job Status  Occupation | Self-Employed | Employer | Formal Employed  (Full time) | Formal Employed  (Part time) | Non-formal  Employed | Other |
| --- | --- | --- | --- | --- | --- | --- |
| Legislator, senior officials and manager | Class I | |  | |  |  |
| Professionals |  |  |  | |  |  |
| Technicians and associate professionals | Class III | Class II | | | |  |
| Clerks |  |  |  |  |  |  |
| Service and sales workers |  |  |  | | |  |
| Skilled agricultural, forestry and fishery | Class V |  | Class VI | | |  |
| Craft and related trades workers | Class III | Class II | Class VI | |  |  |
| Plant, machine operators and assemblers |  |  |  |  |  |  |
| Elementary occupation | Class VI |  | | |  |  |
| Unemployed |  | | | | | Class VI |
|  | | | | | | |

# 
